# Characteristics and Neoplastic Progression in Barrett’s Esophagus: A Large Population-Based Study from Iceland

**DOI:** 10.3390/diagnostics15060684

**Published:** 2025-03-11

**Authors:** Ken Namikawa, Melkorka Sverrisdottir, Hilmar Freyr Fridgeirsson, Hjalti Dagur Hjaltason, Helgi Kristinn Sigmundsson, Jon Gunnlaugur Jonasson, Einar Stefan Bjornsson, Magnus Konradsson

**Affiliations:** 1 Department of Gastroenterology, Landspitali—The National University Hospital of Iceland, 101 Reykjavik, Iceland; 2 Department of Gastroenterology, Cancer Institute Hospital, Japanese Foundation for Cancer Research, Tokyo 135-8550, Japan; 3 Faculty of Medicine, University of Iceland, 101 Reykjavik, Iceland; 4 Department of Pathology, Landspitali—The National University Hospital of Iceland, 101 Reykjavik, Iceland

**Keywords:** Barrett’s esophagus, progression, dysplasia, esophageal adenocarcinoma, epidemiology, Iceland

## Abstract

**Background:** Barrett’s esophagus (BE) is a known precursor to esophageal adenocarcinoma (EAC). However, reports on incidence and progression-to-neoplasm rates have been very variable and conflicting. The aims of the study were to evaluate the characteristics of BE and its progression to neoplasm in a large homogeneous population. **Methods:** This was a retrospective population-based study with patients identified from 11 institutions through the databases in two centralized pathology laboratories. Demographics and relevant clinicopathological features were obtained from medical records among patients with a pathologically confirmed BE by the presence of intestinal metaplasia between 2003 and 2022. **Results:** A total of 1388 patients were identified with BE: 948 were men (69%); the median age at diagnosis was 62 years (IQR, 53–72). The ratio of long-segment BE to short-segment BE was significantly higher in patients ≥ 60 years (1.15, 284/248) than those ≤ 60 years (0.77, 205/265) (*p* = 0.0025). At BE diagnosis, 9.4% had neoplasms: LGD (*n* = 65), HGD (*n* = 16), and EAC (*n* = 49). Among 1258 non-dysplastic BE (NDBE) patients, 4.6% developed a neoplasm—LGD (*n* = 35), HGD (*n* = 8), and EAC (*n* = 15)—with a median observation-period of 5 years (IQR, 3–7). Overall, 160 cases with neoplasms were diagnosed in this BE cohort; 130 (74%) were present at initial BE diagnosis, and 58 (26%) progressed to neoplasms from NDBE. **Conclusions:** The ratio of long-segment BE was found to be significantly higher in patients ≥ 60 years. Around 9% of the patients were diagnosed as harboring a neoplasm concomitantly with BE, accounting for approximately 74% of all neoplasms. After a median follow-up of 5 years, about 5% of BE showed dysplastic or malignant progression.

## 1. Introduction

Esophageal cancer is a disease with a poor prognosis, exhibiting a 5-year survival rate of approximately 20% [1,2]. It is the seventh leading cause of cancer-related deaths worldwide [3]. This high mortality rate is primarily due to the fact that esophageal cancer is often diagnosed at an advanced stage, where curative treatment options are very limited. In its early stages, the disease is generally asymptomatic, making early detection challenging. However, early-stage esophageal cancer can be effectively treated with endoscopic resection or esophagectomy, resulting in a significantly improved 5-year survival rate of approximately 80%. Therefore, early detection is crucial in improving patient prognosis [4,5].

Esophageal cancer is mainly classified into two histopathological subtypes: squamous cell carcinoma and adenocarcinoma. Squamous cell carcinoma is the most common histological type in Asia, the Middle East, Africa, and South America, while adenocarcinoma has become more prevalent in North America and certain European countries [2,6,7,8]. Epidemiological trends indicate that the incidence of squamous cell carcinoma remains stable, whereas the incidence of esophageal adenocarcinoma (EAC) is on the rise [2,6,7,8].

While several risk factors for EAC have been reported, such as male gender, white ethnicity, obesity, smoking, and gastroesophageal reflux disease [8,9,10], Barrett’s esophagus (BE) is of significant clinical interest due to its established role as the only known precursor to EAC [8]. This is characterized by the replacement of the normal squamous epithelium of the esophagus with metaplastic columnar epithelium, a response to chronic esophagitis due to acid reflux. Individuals with BE carry a 30- to 125-fold greater risk of EAC compared to the general population [11].

Based on the reported risk factors, the European Society of Gastrointestinal Endoscopy (ESGE) guidelines state that screening for BE can be considered in patients with long-standing GERD symptoms (i.e., >5 years) and multiple risk factors such as age ≥ 50 years, white race, male sex, obesity, and having a first-degree relative with BE or EAC [12].

BE is more prevalent in Western countries compared to Asia [2,6,8,13,14]; however, significant variations exist even within Western populations regarding its reported prevalence and rates of progression to esophageal adenocarcinoma.

The reported prevalence rates in Western general populations include 1.6% among 1000 adults in Sweden [15] and 1.3% among 1033 adults in Italy [16]. A French nationwide survey study found a 4% prevalence of BE among 2735 individuals who underwent gastroscopy [17]. In a study from the U.S., 6.8% of the people who underwent gastroscopy were diagnosed with BE, with higher rates in those having heartburn (8.3%) compared to those without that symptom (5.6%) [18]. The progression rate from non-dysplastic BE (NDBE) to EAC has been reported to be between 0.12% and 0.8% annually in Western countries [11,19], demonstrating more than a six-fold difference between studies.

The wide variety of reported prevalence of BE and progression-to-neoplasm rates might be related to different risk factors such as race, obesity, and the prevalence of GERD [11,18]. However, it could also be associated with different methodologies used in finding patients, differences in diagnostic criteria, and a lack of diagnosis verification. Identifying the characteristics and progression rates of BE in different regions is crucial in developing effective management strategies based on optimal screening and surveillance, balancing a reduction in disease burden with healthcare resource allocation.

The aim of this study was to evaluate the characteristics of BE and its progression to neoplasm in a large homogenous population-based cohort from Iceland.

## 2. Materials and Methods

### 2.1. Study Design and Study Population

In this retrospective study, individuals aged 18 years and older with pathologically diagnosed BE were analyzed in 11 institutions through two centralized pathology laboratories in Iceland between 1 January 2003 and 31 December 2022.

The potential subjects were identified through searching the topography code T62 (Location: esophagus) in combination with the Systematized Nomenclature of Medicine code M73320 (Diagnosis: intestinal metaplasia) in the pathology database. The definition of BE was based on the European Society of Gastrointestinal Endoscopy guidelines [12], which require the distal esophagus to be lined with columnar epithelium of at least 1 cm in length (tongues or circular) containing intestinal metaplasia (IM) confirmed by histopathological examination. Patients were enrolled according to these criteria, excluding those with inaccurate pathological diagnoses or previous diagnoses of BE before 2003.

The pathology laboratories involved include the Department of Pathology at Landspitali and the Department of Pathology at Akureyri District Hospital. The personal identification numbers of these patients were linked to the electronic medical record system of Landspitali and Iceland’s four regional hospitals in Akranes, Akureyri, Isafjordur, and Neskaupstadur, as well as Husavik hospital, Saudarkrokur hospital, Selfoss hospital, Keflavik hospital, Akureyrar medical clinic, and Vestmannaeyjar hospital.

Clinical, laboratory, endoscopic, and pathological data at the time of initial BE diagnosis and during follow-up for neoplasia progression were collected from medical records. These data include patients’ age at BE diagnosis, gender, length of BE, presence or absence of neoplasm at BE diagnosis, histological type of neoplasm (low-grade dysplasia [LGD], high-grade dysplasia [HGD], or esophageal adenocarcinoma [EAC]), and time for progression to neoplasm. Long-segment and short-segment were adopted as classifications for the length of BE. Maximum extent (M) based on the Prague C&M classification [20] was used to define long-segment (≥3 cm) and short-segment (<3 cm) BE.

### 2.2. Statistical Analysis

Data from initial biopsies confirming IM were used to describe patient and BE characteristics. Descriptive statistics were applied to both continuous and categorical data. Pearson’s chi-squared test, without Yates’ continuity correction, was used to analyze categorical data and compare proportions.

We graphically represented the number of long-segment and short-segment BE cases, the ratio of long-segment to short-segment BE, and the number of NDBE cases, neoplasms cases, and their types (LGD, HGD, or EAC) by gender and age group. The progression time from NDBE, LGD, and HGD was also graphically depicted.

### 2.3. Ethics Approval Statement

This study was approved by the Icelandic Data Protection Authority, the National Bioethics Committee (VSN-23-019), and the director of the scientific committee at Landspitali.

## 3. Results

### 3.1. Study Population and Patient Characteristics

**Figure 1:**
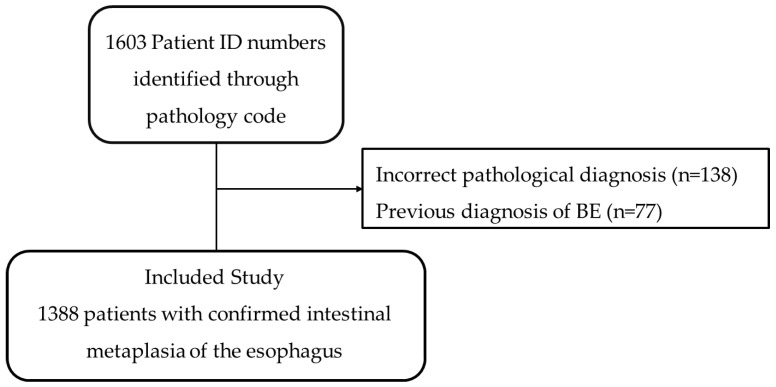
Flow chart of study selection and exclusion process.

**Figure 2:**
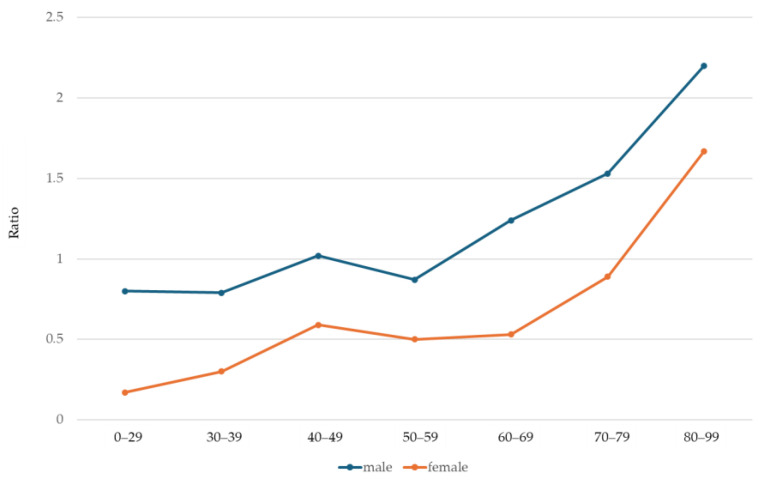
Ratio of the patients with long-segment Barrett’s esophagus to those with short-segment Barrett’s esophagus by age group. This was calculated by dividing the number of patients with long-segment Barrett’s esophagus (BE) by those with short-segment BE in each age group.

Overall, 1388 BE patients were identified with confirmed IM out of a total of 1603 patients retrieved using the pathology codes, excluding those with inaccurate pathological diagnoses (*n* = 138) or a prior BE diagnosis before 2003 (*n* = 77) (Figure 1). Out of 1388 in the study population, 948 (68%) were males and 440 (32%) were females, with a median age of 62 years (IQR, 53–72). Among 1002 patients with available BE length information, 489 (49%) had long-segment BE and 513 (51%) short-segment BE (Table 1). The ratio of the number of patients with long-segment BE to those with short-segment BE is significantly higher in males (long-segment: 380 vs. short-segment: 337) than in females (long-segment: 109 vs. short-segment: 176) (*p* < 0.0001). As shown in Figure 2, this ratio positively correlated with age in both genders, showing a higher ratio in males across all age groups. This ratio was also significantly higher in patients aged 60 years or older (long-segment: 284 vs. short-segment: 248) compared to those younger than 60 (long-segment: 205 vs. short-segment: 265) (*p* = 0.0025). Detailed numbers of long-segment and short-segment BE patients by gender and age group are presented in Supplementary Figure S1.

### 3.2. Neoplasm at Initial BE Diagnosis

**Figure 3:**
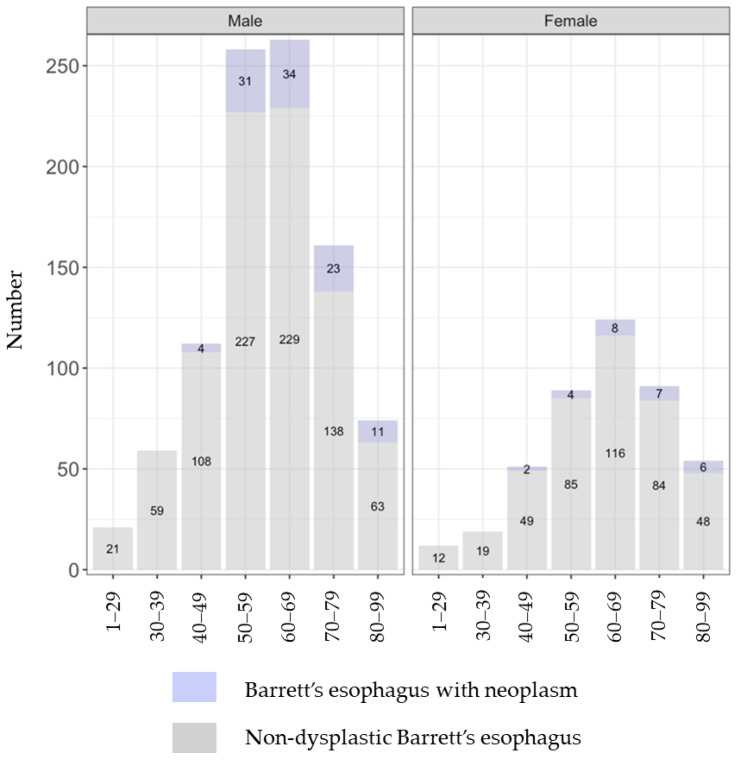
The distribution of the patients with and without neoplasm at initial diagnosis of Barrett’s esophagus. They are shown here by age group and gender.

**Figure 4:**
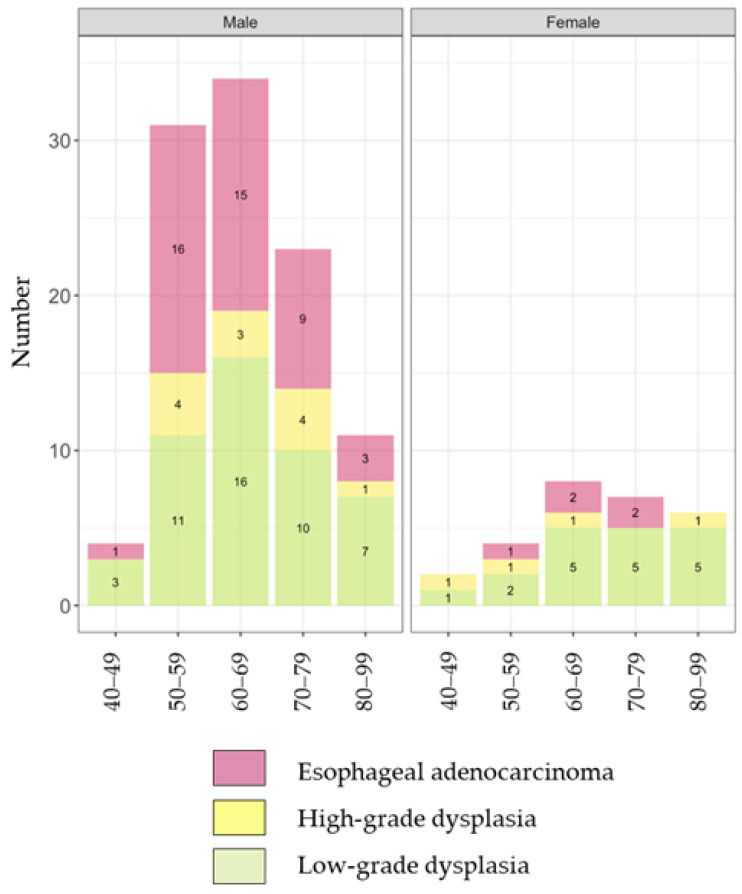
Distribution of the type of neoplasm at initial diagnosis of Barrett’s esophagus by age group and gender.

At the initial diagnosis of BE, 9.4% (130/1388) of patients were diagnosed concomitantly with a neoplasm (Table 1). The distribution by age group and gender of those with a concomitant neoplasm at BE diagnosis is shown in Figure 3. None of the patients under 40 years old had a concomitant neoplasm. The detected neoplasms at initial BE diagnosis included 65 cases of LGD (4.7%), 16 cases of HGD (1.2%), and 49 cases of EAC (3.5%) (Table 1). The numbers of the different types of neoplasms by age group and gender are detailed in Figure 4.

### 3.3. Progression to Neoplasm

**Figure 5:**
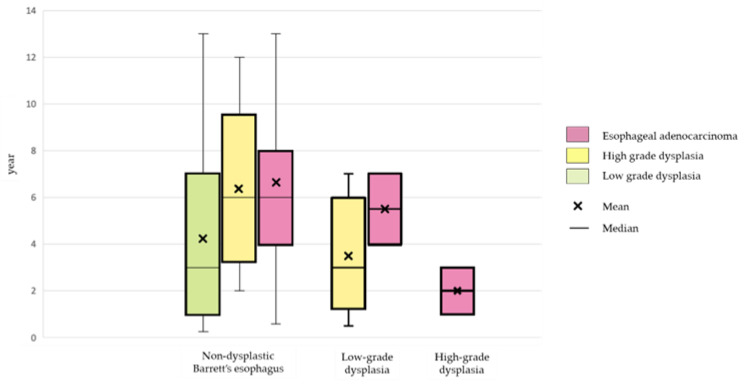
The progression time to neoplasm from each initial status at diagnosis with Barrett’s esophagus.

Table 2 presents the number of patients who experienced tumor progression from NDBE, LGD, and HGD. Among NDBE patients, 4.6% (58/1258) developed neoplasms over a median follow-up period of 5 years (IQR, 3–7). Specifically, 2.8% (35/1258) progressed to LGD, 0.6% (8/1258) to HGD, and 1.2% (15/1258) to EAC over median follow-up periods of 3 (1–7), 6 (3.25–9.5), and 6 (4–8) years, respectively (Figure 5). In patients with LGD at initial BE diagnosis, 7.7% (5/65) progressed to HGD, 3.1% (2/65) to EAC, and 89.2% (58/65) did not progress (Table 2). The median progression time from LGD to HGD was 3 (IQR, 1.25–6) years, and 5.5 (IQR, 4–7) years to EAC (Figure 5). In patients with HGD at initial BE diagnosis, 12.5% (2/16) progressed to EAC during a median follow-up time of 2 (IQR, 1–3) years (Figure 5), whereas 87.5% (14/16) did not progress (Table 2).

### 3.4. Characteristics of EAC

The clinical characteristics of patients with EAC in BE are demonstrated in Supplementary Table S1. The median age at diagnosis with EAC was 66 (IQR, 59–73). Out of the patients with EAC in BE, 91% (62/68) were males, and 85% (41/48) demonstrated long-segment BE. EAC was concomitantly diagnosed at BE diagnosis in 49 (72.1%) patients and progressed from HGD in 2 (2.9%), LGD in 2 (2.9%), and NDBE in 15 (22.1%).

## 4. Discussion

The results of the current study, which is the first analysis of BE epidemiology in Iceland, highlight the features of pathologically confirmed BE and its progression to tumors in a relatively large, homogenous, predominantly Caucasian population over a 20-year period.

One of the strengths of this study lies in the collection of BE data in a well-organized cohort based on diagnosis with IM through two centralized pathology laboratories, which cover two thirds of pathology laboratories in Iceland. In contrast, pathological confirmation is not essential for diagnosis of BE in some guidelines (endoscopic diagnosis is also adopted) [21,22], and the presence of IM is not essential (gastric metaplasia is adopted, as well as IM) [23,24] in other guidelines. Mango P et al. found that their study on BE had a methodological limitation as the subjects were included without correlating description of the biopsy site with endoscopic findings. This limitation could cause uncertainty in BE diagnosis since biopsy near the squamous-columnar junction might reveal IM in the cardia of the stomach [25]. These patients were excluded from the current study after we reviewed the endoscopic findings and pathological descriptions, considering the risk of incorrect pathological diagnosis. While our cohort consisted of patients from 11 institutions, the histopathological diagnosis of BE and neoplasms was relatively standardized since only two centralized pathology laboratories were involved. According to previous studies, intra-observer discordance has been demonstrated among pathologists in the diagnosis of neoplasms in BE [26,27,28]. Furthermore, the current study population was based on comprehensive data collection for patients receiving follow-up surveillance over a long period of time. This was enabled by linking national identification numbers for individuals in Iceland to the electric medical record system in the whole country.

The male predominance (69%) and median age of 62 years at diagnosis reported in our study are in line with other studies, indicating that BE is typically diagnosed in male adults in their early 60s [11,29,30,31]. In the current study, the ratio of patients with long-segment BE to those with short-segment BE was positively correlated with the age group. Interestingly, this finding contrasts with recent reports suggesting that BE is fully developed by the time of index endoscopy [32] and that the percentage of long-segment BE does not vary between generations [30,32]. However, previous studies have shown variable results. Some studies have demonstrated that the length of BE can vary, as antacids or surgical interventions can shorten Barrett’s esophagus by reducing acid reflux [33,34,35]. Iftikhar et al. also observed a non-significant trend towards an increasing length of BE (mean initial length of 6.9 cm vs. mean last length of 7.6 cm) over a mean follow-up period of 54 months [31]. Furthermore, when focusing on patients who developed dysplasia, the length of the BE significantly increased during observation compared to those who did not develop dysplasia [31]. Our results that more patients have long-segment BE in an older age group might be in agreement with the observation above; an accumulated increase in length of BE over decades is shown as a significant difference between young and old age groups (the ratio of the number of patients with long-segment BE to those with short-segment BE; 0.56 in the 0–29 year group vs. 2.0 in the 80–99 year group). Alternatively, the elderly population diagnosed at the initial phase of the study may have experienced poorly controlled acid reflux at a younger age, leading to longer segments of BE due to the fact that those individuals might have been exposed to acid reflux in the era prior to proton pump inhibitors (PPIs), which arrived on the market in 1988.

In the present study, 9.4% of the subjects had already developed neoplasms at initial BE diagnosis, accounting for 74% of all neoplasm cases. EAC accounted for 38% of these detected neoplasms at BE diagnosis. Given these rates, it may be beneficial to consider performing gastroscopy for screening at an earlier age compared to the current practice in Iceland. Furthermore, the rate of EAC out of the neoplasms concomitantly detected with BE was particularly high (52%) in males in their 50s, and more than half of these EAC cases were diagnosed with cancer at stage pT2 or higher. On the other hand, no patient under 40 years old developed a neoplasm, regardless of gender. In terms of the age to consider BE screening, aged ≥50 years is suggested in the ESGE guidelines [12], and aged >50 years is suggested in the American Gastroenterological Association (AGA) guidelines [36] and the American College of Gastroenterology (ACG) guidelines [37]. Given the variations in the prevalence and progression rates of BE, it may be necessary to develop guidelines for screening tailored to the specific situation in each country or area. While this study does not address the necessity of screening for the general population in terms of cost-effectiveness, it offers valuable insights into the optimal age for screening within the targeted cohort. For instance, based on our results, the first screening gastroscopy to detect BE and initiate surveillance for detecting tumors at the stage of dysplasia does not have to be carried out in people under 40 years; however, it may be recommended completing at the age of 50 in Iceland. Since long-segment BE, which carries a high risk of developing EAC, was significantly more prevalent among individuals aged ≥60 years in the present study, screening may be intensively recommended for this age group. Thus, our findings provide a crucial baseline for understanding the incidence and progression of BE in Iceland and implicated the potential necessity of conducting tailored screening programs, underscoring the importance of understanding local epidemiology in different populations.

In the present BE cohort, the cumulative incidence of EAC was 4.9%, which aligns with the results of a reported meta-analysis indicating that approximately 3% to 5% of patients with BE will be diagnosed with EAC in their lifetime [38]. The male predominance (91%), median age of 66 years, and predominance of long-segment BE in patients who developed EAC were consistent with previous reports [30,39]. The median time for NDBE to progress to LGD, HGD, and EAC in our data was 3, 6, and 6 years, respectively. These results are also compatible with previous reports. Wani S et al. reported a mean time for NDBE of 5.3 years (range, 1.05–15.3) to develop EAC and 5.6 years (range, 1.15–18.66) to develop HGD [40].

The current study showed a 1.2% cumulative incidence of developing EAC from NDBE with a median observation period of 6 years. This is consistent with recently reported results of 0.1% to 0.48% [11,40,41,42] for the annual rate of developing EAC for NDBE patients, which are lower than those previously reported [19,43].

The present study has several limitations. Firstly, it is a retrospective and observational study, which may lead to some biases and low data quality. There are some missing data on clinical information due to the varying accuracy of data description among endoscopists, which made risk factor analysis impossible due to limited information on lifestyle, GERD symptoms, and PPI use. Secondly, the identification of the study population relied on biopsy specimens examined only in the patients who underwent gastroscopy on indication. Thus, it is not entirely representative of the general population. Thirdly, methodological differences in biopsy protocols for BE may have existed among endoscopists; the ability to detect neoplasm and compliance with the Seattle protocol varied among doctors and periods. Hence, it is uncertain whether an adequate number of systematic endoscopic biopsies were performed during endoscopy, leading to a potentially underestimated number of BE and neoplasms cases.

To improve patient outcomes and optimize healthcare resource allocation, future research should preferably be based on results from prospective studies, validating the findings of this study and providing more accurate incidence and progression-to-neoplasm rates in BE. Furthermore, there is a need to investigate the impact of various risk factors on progression to EAC, which may help in developing tailored screening and surveillance strategies.

## 5. Conclusions

The results of the present study provide valuable insights into the epidemiology of Barrett’s esophagus. While gender distribution, common age for disease, and progression to EAC from NDBE were consistent with previously published studies, the proportion of long-segment BE was significantly higher in individuals ≥ 60 years. We found that around 9% of the study population had already developed neoplasms at the initial BE diagnosis, accounting for approximately 74% of all neoplasms.

Considering the rate of EAC, which accounted for 38% of these concomitant neoplasms at BE diagnosis, with a particularly high rate of 52% in males in their 50s, it may be worth considering performing gastroscopy for screening at an earlier age compared to the current practice in Iceland, as it is suggested that there are potential benefits in completing the first screening by the age of 50 years. Thus, we can highlight the importance of understanding local epidemiology in different populations; however, further prospective studies are needed to validate our findings.

## Figures and Tables

**Table 1:** Clinical characteristics of the patients.

Characteristics	(*n* = 1388)
Age (years)	
Median (IQR), years	62 (53–72)
Sex	
Male	948 (68.3%)
Female	83 (31.7%)
Length of BE	
Long-segment	489 (48.8%)
Short-segment	513 (51.2%)
NA	386
Neoplasm at BE diagnosis	
EAC	49 (3.5%)
HGD	16 (1.2%)
LGD	65 (4.7%)
NDBE	1258 (90.6%)

IQR; interquartile range, BE; Barrett’s esophagus, EAC; adenocarcinoma, HGD; high-grade dysplasia, LGD; low-grade dysplasia, NDBE; non-dysplastic Barrett’s esophagus.

**Table 2:** Tumor progression from NDBE, LGD, and HGD.

	NDBE (*n* = 1258)	LGD (*n* = 65)	HGD (*n* = 16)
Progression to neoplasm			
EAC	15 (1.2%)	2 (3.1%)	2 (12.5%)
HGD	8 (0.6%)	5 (7.7%)	-
LGD	35 (2.8%)	-	-
No progression	1200 (95.4%)	58 (89.2)	14 (87.5%)

NDBE; non-dysplastic Barrett’s esophagus, LGD; low-grade dysplasia, HGD; high-grade dysplasia, EAC; adenocarcinoma.

## Data Availability

The original contributions presented in this study are included in the article. Further inquiries can be directed to the corresponding author.

## Supplementary Materials

The following supporting information can be downloaded at: https://www.mdpi.com/article/10.3390/diagnostics15060684/s1, Figure S1. Distribution of the patients with long-segment and short-segment Barrett’s esophagus by age group and gender. Table S1. Clinical characteristics of the patents with EAC in BE.
