# Supplementary material for: Characteristics and Neoplastic Progression in Barrett’s Esophagus: A Large Population-Based Study from Iceland
Source: Diagnostics (Basel). 2025 Mar 11;15(6):684. doi: 10.3390/diagnostics15060684 (PMC11941158; doi:10.3390/diagnostics15060684)
Supplement: Supplementary file 1 [file diagnostics-15-00684-s001.zip › diagnostics-3509251-SI.pdf]

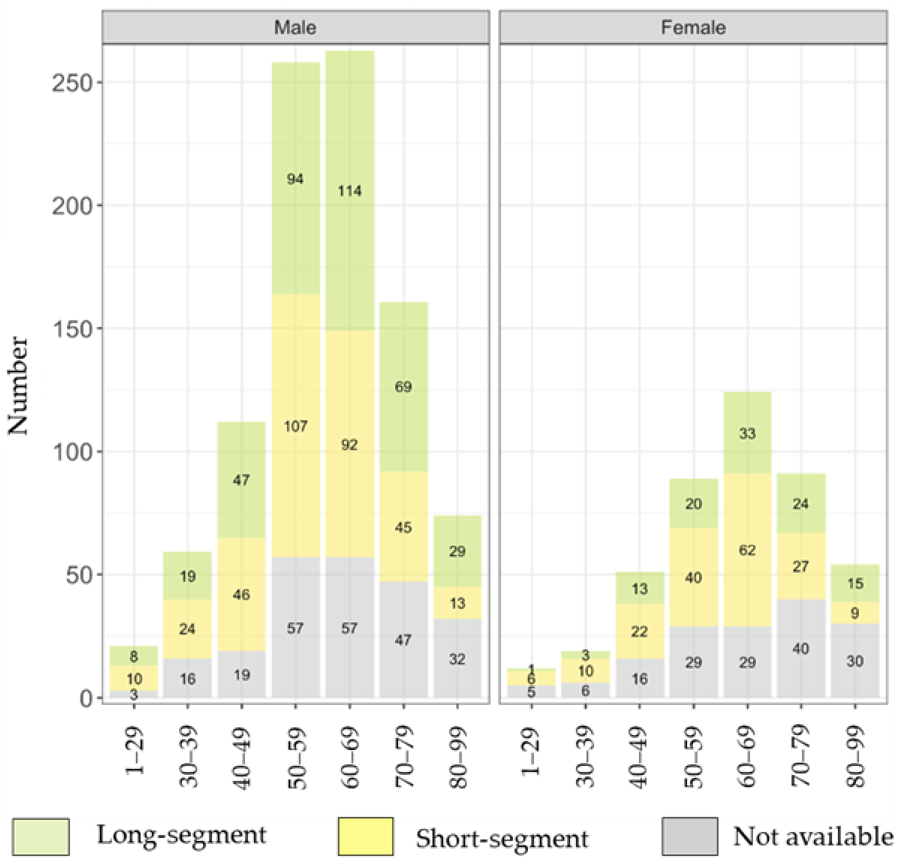


**Supplementary Figure S1.** Distribution of the patients with long-segment and short-segment Barrett's esophagus by age group and gender.

**Supplementary Table S1.** Clinical characteristics of the patients with EAC in BE

| **Characteristics** | (n=68) |
|---|---|
| Age (years) | |
| Median (IQR), years | 65.5 (59-72.5) |
| Gender | |
| Male | 62 (91.2%) |
| Female | 6 (8.8%) |
| Length of BE | |
| Long-segment | 41 (85.4%) |
| Short-segment | 7 (14.6%) |
| NA | 20 |
| Neoplasm at BE diagnosis | |
| Concomitantly diagnosed at BE-diagnosis | 49 (72.1%) |
| Progressed from HGD | 2 (2.9%) |
| Progressed from LGD | 2 (2.9%) |

| | |
|---|---|
| Progressed from NDBE | 15 (22.1 %) |

EAC; adenocarcinoma, BE; Barrett's esophagus, IQR; interquartile range, HGD; high-grade dysplasia, LGD; low-grade dysplasia, NDBE; non-dysplastic Barrett's esophagus
